# Current Research Landscape of Marine-Derived Anti-Atherosclerotic Substances

**DOI:** 10.3390/md18090440

**Published:** 2020-08-25

**Authors:** Qi Cao, Jiarui Zhao, Maochen Xing, Han Xiao, Qian Zhang, Hao Liang, Aiguo Ji, Shuliang Song

**Affiliations:** 1 Marine College, Shandong University, Weihai 264209, China; sddxcqq@163.com (Q.C.); 201936684@mail.sdu.edu.cn (J.Z.); sddxxmc@163.com (M.X.); 15651795075@163.com (H.X.); zhangqianzq@sdu.edu.cn (Q.Z.); lianghao@sdu.edu.cn (H.L.); 2 School of Pharmaceutical Sciences, Shandong University, Jinan 250012, China

**Keywords:** atherosclerosis, marine active substances, polysaccharides, proteins and peptides, polyunsaturated fatty acids, small molecule compounds

## Abstract

Atherosclerosis is a chronic disease characterized by lipid accumulation and chronic inflammation of the arterial wall, which is the pathological basis for coronary heart disease, cerebrovascular disease and thromboembolic disease. Currently, there is a lack of low-cost therapeutic agents that effectively slow the progression of atherosclerosis. Therefore, the development of new drugs is urgently needed. The research and development of marine-derived drugs have gained increasing interest from researchers across the world. Many marine organisms provide a rich material basis for the development of atherosclerotic drugs. This review focuses on the latest technological advances in the structures and mechanisms of action of marine-derived anti-atherosclerotic substances and the challenges of the application of these substances including marine polysaccharides, proteins and peptides, polyunsaturated fatty acids and small molecule compounds. Here, we describe the theoretical basis of marine biological resources in the treatment of atherosclerosis.

## 1. Introduction

The morbidity, mortality and disability rates of cardiovascular and cerebrovascular diseases (CVDs) are the highest in the world and have a major negative impact on human health and quality of life. Atherosclerosis (AS) is significant pathological basis of CVDs. Research shows that the occurrence of AS is the result of a combination of multiple factors. There are many risk factors for AS that have been discovered, including hyperlipidemia, high levels of low-density lipoprotein cholesterol (LDL-C), hypertension, smoking, diabetes and obesity [1]. However, the available management strategies are inadequate and include the adjustment of blood lipid levels and anti-inflammatory treatments. Statins are currently the most effective lipid-lowering drugs and significantly reduce LDL-C and stabilize plaques. Recent reports indicate that certain statins may cause undesirable side effects such as myopathy [2,3]. Therefore, there is increasing interest in alternative or complementary therapies for AS that can effectively treat the disease without any side effects [4]. Due to their unique growth environment, marine organisms have formed natural active substances with novel structures and unique functions during the long evolutionary process.

In this review, we summarized the latest research on marine-derived active substances that have a positive therapeutic effect on AS including polysaccharides, proteins and peptides, polyunsaturated fatty acids and small molecule compounds. For each active substance, we discussed its structure and therapeutic effects on atherosclerotic plaques. In addition, we further outlined the possible mechanisms by which these active substances exert their anti-atherosclerotic effects. This review provides a theoretical basis for the in-depth study of marine-derived active substances in the treatment of AS.

## 2. Polysaccharides

### 2.1. Fucoidan

**Figure 1:**
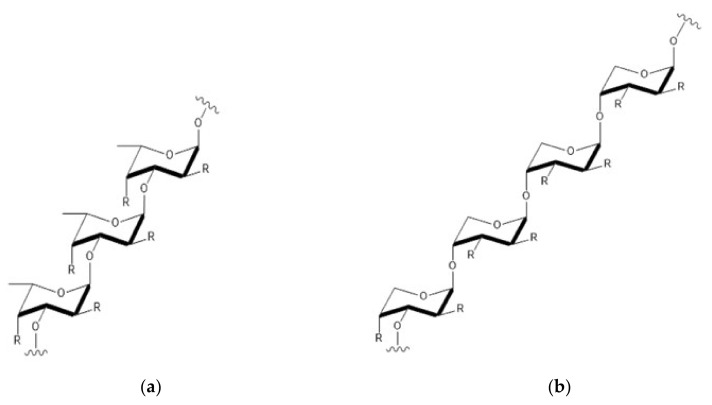
The two main chain structures in fucoidan (simplified). (**a**): Structure of type 1 fucoidan molecules with (1→3)-α-l-fucopyranose. (**b**): Structure of type 2 fucoidan molecules with α-l-fucopyranose linked by (1→3) and (1→4). The ‘R’ can be a monosaccharide or a sulfate group [12].

As a natural polysaccharide derived from the ocean, fucoidan is mainly extracted from brown algae and some marine invertebrates [5,6]. Fucoidan is a water-soluble polysaccharide, which composed of L-fucose and sulfate groups. Its main monosaccharide component is L-fucose-4-sulfate and contains lessglucuronic acid, mannose, rhamnose, glucose, arabinose and xylose [7,8]. Affected by seaweed species, extraction methods, seasons, regions and other factors, the composition and chemical structure of fucoidan are complex and changeable [9]. There are two main chain structures in fucoidan, one with (1→3)-α-l- fucopyranose and the other with α-l-fucopyranose linked by (1→3) and (1→4) (Figure 1). Some fucoidans possess substituted branches at the C-2 and C-3 positions and fucosyl residues are usually sulfated at the C-2 and/or C-4 position [10,11].

Fucoidan decreases the area of atherosclerotic plaques. It has been reported that apolipoprotein E-deficient (ApoE-/-) mice receiving an atherogenic diet with fucoidan show ameliorated atherosclerotic lesions in the aortas in a dose-dependent manner [13,14]. Additionally, spontaneously hyperlipidemia mice received high-fat diet supplemented with fucoidan exhibited inhibition in the development of AS [15]. The composition and effect of fucoidans are summarized in Table 1.

Dyslipidemia is an independent risk factor of AS [16]. Studies have shown that fucoidan lowers blood lipids and corrects abnormal lipid metabolism by regulating various target genes and proteins. Fucoidan from brown algae has been demonstrated to reduce the levels of total cholesterol (TC), triacylglycerol (TG) and LDL-C in the serum of mice and rats and increase high-density lipoprotein cholesterol (HDL-C) levels [15,17,18,19]. Similarly, fucoidan from sea cucumber lowers the levels of TC and LDL-C and reduces the weight of adipose tissue in mice [20,21]. In addition, low molecular weight fucoidan diminishes TG and oxidized low-density lipoprotein (ox-LDL) levels, leading to stabilization of established atherosclerotic lesions [14]. The liver is an important organ participating in reverse cholesterol transport (RCT). RCT is believed to attenuate the progression of AS and effectively regulates cholesterol synthesis and lipid metabolism. In the liver of high-fat diet-fed mice, fucoidan supplementation improves the level of lipid metabolism-related genes such as peroxisome proliferator-activated receptor alpha (PPARα) and gamma (PPARγ), liver X receptor (LXR) alpha, ATP-binding cassette transporter A1 and G8 and scavenger receptor B type 1 (SR-B1), thereby increasing the initial level of RCT, promoting lipid absorption and metabolism and reducing TG levels [15,22,23,24].

An inflammatory response is present during the formation of atherosclerotic plaques and high levels of inflammatory cytokines in the serum have been used as an important predictor for AS. Recent studies have shown that fucoidan treatment reduces inflammatory cytokines in RAW264.7 macrophages and mice [25,26,27,28,29]. Low molecular weight fucoidan regulates the production and expression of inflammatory cytokines including IL-6, IL-10, phosphorylated c-Jun N-terminal kinase(p-JNK) and cyclin D1 and inhibits the inflammation associated with AS [14]. Fucoidan treatment decreases the expression of lectin-like ox-LDL receptor-1 (LOX-1) and the levels of pro-inflammatory cytokines 1L-1β, IL-6, tumor necrosis factor-alpha (TNF-α), vascular cell adhesion molecule-1 (VCAM-1),and intercellular cell adhesion molecule-1 (ICAM-1) in low-density lipoprotein receptor-deficient (LDLR-/-) mice [25], suggesting that fucoidan reduces AS in LDLR-/-mice by inhibiting the inflammatory response. Inhibition of nuclear factor-kappa B (NF-κB) and mitogen-activated protein kinase (MAPK) is also considered an effective approach to treat inflammatory diseases [30,31]. Studies have shown that fucoidan inhibits the pro-inflammatory cytokines IL-6 and TNF-α, nitric oxide (NO) and prostaglandin-E2 in RAW264.7 macrophages stimulated by lipopolysaccharide (LPS) in a dose-dependent manner, suggesting that fucoidan may inhibit NF-κB and MAPK pathways to reduce the production of inflammatory mediators and pro-inflammatory cytokines [29].

The platelet is a major player in initiation of the atherosclerotic process. The initial stage of atherosclerotic plaque development is that platelets activate endothelial cells and adhere to them [32]. Selectins are necessary to promote the rolling and adhesion of platelets and leukocytes [33,34]. Among them, P-selectin can promote the adhesion of leukocytes, which helps thrombus growth [35]. L-selectin can mediate leukocytes rolling and causes neutrophils to adhere to inflamed endothelial cells [36]. Fucoidan can be used as a ligand for P-selectin and L-selectin, thereby inhibiting the activity of selectin, showing promise as an anti-inflammatory agent for the treatment of AS in the early stage [34,37,38,39]. In addition, the anti-inflammatory effect of fucoidan may be related to the competitive blocking of macrophage scavenger receptors A (SR-A) by fucoidan as a ligand [40]. Fucoidan combined with SR-A can induce protein tyrosine phosphorylation of protein kinases, protein kinase C (PKC) activity and specifically stimulate the activity of p21-activated kinase, MAPK extracellular signal-regulated kinase (ERK), JNK and p38 MAPK, leading to the secretion of inflammatory cytokines and increased levels of urokinase-type plasminogen activator [41,42]. It has been proved that fucoidan can induce the production of nitric oxide through SR-A, which may be mediated by p38 mitogen-activated protein kinase and NF-κB-related signaling pathways [43]. It is worth noting that, in addition to fucoidan, other ligands of SR-A, such as ox-LDL, acetylated LDL, maleylated bovine serum albumin and dextran sulfate, did not produce this phenomenon [43]. Therefore, the signal transduction pathway mediated by SR-A is affected by the characteristics of the ligand. However, the results of research in this area are limited and more studies are needed to clarify the influence of the combination of fucoidan and SR-A on the anti-inflammatory effect of fucoidan. Reactive oxygen species (ROS) play a significant role in the development of cardiovascular diseases [44,45]. The inhibitory effect of fucoidan on AS may be related to its antioxidant activity [46] and studies have shown that fucoidan treatment dampens ROS generation as well as the expression of ROS generation-related proteins in the aorta of LDLR-/-mice including endothelial nitric oxide synthase (eNOS), superoxide dismutase 1 (SOD1) and NADPH oxidase subunit 2/4 (NOX-2/4) [25] Fucoidan partly restores the lipid peroxidation and antioxidant protection system in a murine model of alimentary hyperlipidemia [18].

In conclusion, several studies have shown that fucoidan is a promising anti-atherosclerotic drug by activating multiple signaling pathways that regulate lipid metabolism, suppress inflammation and oxidative stress.

### 2.2. Alginate

**Figure 2:**
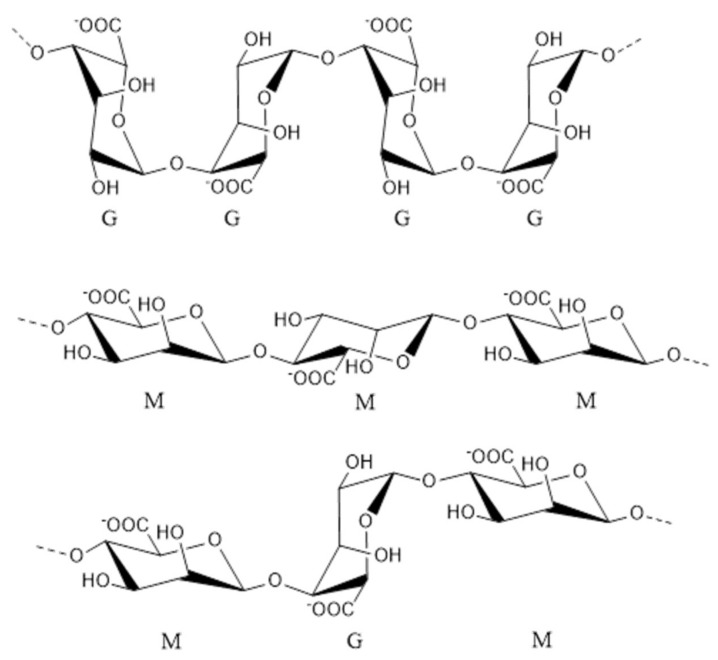
Chemical structures of G-block, M-block and alternating block in alginate [51].

Alginate is mainly derived from brown seaweed and is composed of β-d-mannuronic acid and α-l-guluronic acid monomers [47], which are linked by 1,4-O-glycosidic bonds. Alginate occurs in nature as poly-a-l-guluronate (PG), poly-b-d-mannuronate (PM) or a heteropolymer form (Figure 2) [48,49,50,51]. Recently, alginate oligosaccharide, which is a degradation product of alginate, has attracted increasing attention due to its excellent solubility in water, small molecular weight and biological activities [52,53,54,55,56,57,58,59].

Several studies reported that alginate treatment effectively relieves hyperlipidemia and reduces the probability of cardiovascular disease such as AS [56,60]. Obese mice induced by high-fat diets show decreases in body weight, fat accumulation and serum TG and TC after receiving sodium alginate for four weeks [61]. In another study, rats were given a calcium-alginate (Ca-Alg) diet for two weeks, the plasma cholesterol and bile acid levels in portal vein plasma were significantly reduced and the bile acid content in feces increased. The possible mechanism is that the combination of Ca-Alg and bile acid inhibits the reabsorption of bile acid, thereby stimulating the synthesis of bile acid from cholesterol in the liver, leading to a decrease in plasma cholesterol concentration. [56].

Both PM and PG show the potential to reduce serum cholesterol levels in rats and alginate rich in mannuronic acid has been demonstrated to achieve a better effect on reducing liver cholesterol levels [60]. It is worth noting that chemically-modified alginate shows higher lipid-lowering activity. For example, amidated sodium alginate has the effect of reducing rat serum cholesterol, TG, liver cholesterol and liver total lipids. However, natural sodium alginate can only lower liver cholesterol levels but does not affect the levels of serum cholesterol, TG and liver total lipids in rats. The high activity of amidated alginate in reducing cholesterol and blood lipids can be attributed to its promotion of the excretion of cholesterol and coprostanol in feces [62].

Hepatic LDLR plays a key role in lipoprotein metabolism by reducing the plasma LDL-C concentrations, which reduces the risk for cardiovascular diseases [63]. Studies have found that alginate oligosaccharides can increase the expression of LDLR and the uptake of LDL in liver cells in a dose- and time-dependent manner. Moreover, this effect is dependent on sterol responsive element binding protein-2 (SREBP-2) and proprotein convertase subtilisin/kexin type 9 [64,65].

### 2.3. Ulvan

**Figure 3:**
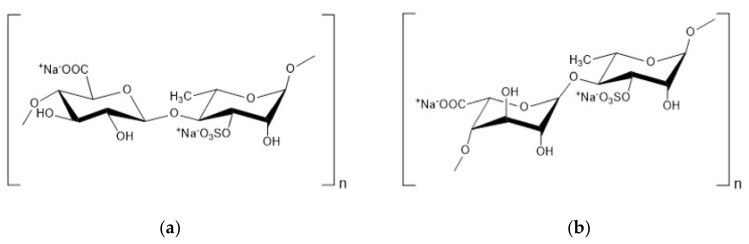
Chemical structures of two main repeating disaccharides in ulvan from *Ulva rigida.* (**a**): sodium ulvanobiuronate 3-sufate A (A3S). (**b**): sodium ulvanobiuronate 3-sufate B (B3S) [68].

Ulvan is a natural sulfated heteropolysaccharide derived from green algae that is mainly extracted from *Ulva lactuca* and *Ulva pertusa* [66,67]. Ulvan consists of the disaccharide units d-glucuronic acid or l-iduronic acid attached through a sulfated 1,4 l-rhamnose residue with traces of d-xylose and d-glucose. The main disaccharide units are β-d-Glcp A-(1→4)-α-l-Rhap 3S and α-l-Idop A-(1→4)-α-l-Rhap 3S, which are called the ulvanobiuronic acid 3-sulfate type A or type B and symbolized by A3S or B3S, respectively (Figure 3) [68,69].

The anti-hyperlipidemia activity of ulvan makes it a prime candidate for the treatment of AS. Treating hamsters fed with a high-cholesterol diet with ulvan derived from *Ulva rigida* for 12 weeks reduces the aortic fatty streak area by 70% [70]. Similar treatments in rats with ulvan derived from *Ulva lactuca* induces decreases in serum total lipids, TC, TG and LDL-C by 61%, 49.6%, 66% and 93%, respectively. In addition, ulvan also decreases very-low-density lipoprotein cholesterol (VLDL-C). Subsequently, HDL-C concentration is markedly increased by 180% and the atherogenic index of hypercholesterolemic rats is reduced by 94% [71]. In another study, ulvan derived from *Ulva pertusa* leads to a decrease in TC, TG and LDL-C levels and an increase in HDL-C levels [72]. Therefore, it has been proved that ulvan from various sources can improve animal blood lipid levels, reduce TC, TG, LDL-C, VLDL-C and increase HDL-C levels which may have a positive therapeutic effect on AS [70,71,72,73,74,75].

The molecular weight and sulfate content of ulvan affects its activity on lipid metabolism. High molecular weight ulvan is effective for reducing serum TC and LDL-C whereas low molecular weight ulvan impacts TG and HDL-C [76]. Moreover, low molecular weight ulvan usually has better antioxidant activity [77]. In another study, the activity of ulvan containing high amount of sulfates on TG and LDL-C is more pronounced compared with natural ulvan [73]. Mechanistic studies showed that high sulfate ulvan downregulates LXR and upregulates farnesoid X receptor and PPARγ to improve lipid profiles in hyperlipidemia rats [74]. The composition and effect of ulvan are summarized in Table 2.

### 2.4. Enteromorpha Prolifera Polysaccharides

*Enteromorpha prolifera* (*E. prolifera*) is a common green algae [78]. *E. prolifera* polysaccharides (EPs) mainly consists of rhamnose, uronic acid, glucose, xylose and sulfate [79]. The backbone of EPs consisted of d-GlcUA p-α-1,4-3-sulfate-l-Rha p-β-1,4-d-Xyl p-β-1,4-3-sulfate-l-Rha p units [80]. The composition and effect of *E. prolifera* polysaccharides are shown in Table 3.

Studies have demonstrated the hypolipidemic activity of EPs in rats and mice in a dose-dependent manner [78]. One study report that EPs inhibits the increase in body weight, decreases liver weight and plasma LDL-C, TG and TC levels and increases HDL-C in rats fed with high-fat diets [81]. Previous mechanistic studies have shown that EPs suppresses sterol regulatory SREBP-2, which is a key transcription factor in cholesterol metabolism and regulates the expression of 3-hydroxy-3-methylglutaryl coenzyme A reductase (HMGCR) to reduce serum TC levels [82]. In addition, EPs downregulates the expression of acetyl-CoA carboxylase (ACC) by inhibiting sterol regulatory element-binding protein-1c (SREBP-1c), thereby reducing serum TG levels [83].

EPs also shows excellent antioxidant activity. EPF2 obtained from the crude polysaccharides of *E. prolifera* increase the level of endogenous antioxidant enzymes, including glutathione peroxidase (GSH-Px), catalase (CAT) and superoxide dismutase (SOD) in mice received high-fat diet and reduces the content of malondialdehyde (MDA) in serum [78]. These results suggest that EPs may reduce the risk of hyperlipidemia, thereby inhibiting the risk of cardiovascular and cerebrovascular diseases such as AS [84,85].

### 2.5. Porphyra Polysaccharides

The main components of porphyra are polysaccharides, proteins, vitamins, fatty acids and minerals [86]. Porphyra is one of the seaweeds with rich polysaccharide content, which is usually between 20% and 40% and varies with the type, location and growth time of seaweed [87]. It has been reported that the structure of sulfated polysaccharides isolated from *Porphyra haitanensis* has alternately connected 3-linked β-D-galactosyl units and 4-linked a-l-galactosyl 6-sulfate and 3,6-anhydro-a-l-galactosyl units [88,89].

Polysaccharides extracted from porphyra are reported to have anti-hyperlipidemia activity. Li et al. found that polysaccharides obtained from *Porphyra yezoensis* reduces plasma and liver TG and TC in rats fed with a high-fat diet. Furthermore, plasma LDL-C is decreased and plasma HDL-C is increased paired with reductions in liver weight [90]. Polysaccharides extracted from *Porphyra haitanensis* also have similar properties, which decreases TC, TG and LDL-C levels by 28.5%, 29.4% and 33.5%, respectively, in mice fed with a high-fat diet [89].

Studies have shown that porphyra polysaccharides could be used as natural Therapeutic agent in preventing the hyperlipidemia and this effect may be attributed to its excellent antioxidant potential [89]. Porphyra polysaccharides increase the content of SOD, GSH-Px and CAT in mice and reduce the level of MDA [88,89,91]. Simultaneously, Porphyra polysaccharides also reduce the NO level to avoid lipid peroxidation [89].

### 2.6. Chondroitin Sulfate

**Figure 4:**
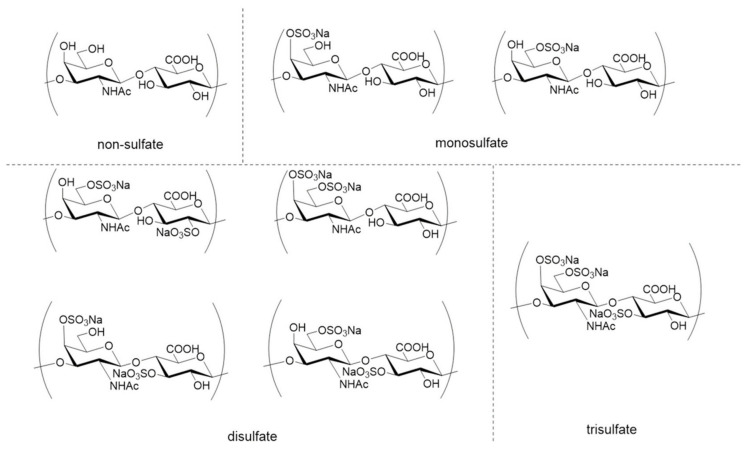
Structure diversity of chondroitin sulfates with various sulfation patterns [92].

As a natural glycosaminoglycan, chondroitin sulfate (CS) is constituted by repeating disaccharides of D-glucuronic acid and *N*-acetyl-D-galactosamine. The resulting repeating disaccharide unit →4-β-GlcA-(1→3)-β-GalNAc-1→ can be sulfated to various extents, which is shown in Figure 4 [92]. The presence of CS has been found in marine organisms such as shark, squid, sea cucumber, skate and sturgeon [93,94,95,96,97].

According to reports, CS effectively relieves the progression of AS. ApoE-/-mice treated with CS for 4 weeks exhibit attenuated atherosclerotic lesions [98]. Studies have shown that patients with rheumatoid arthritis (RA) suffer from AS earlier than normal people and atherosclerotic plaques progress faster [99,100]. Studies have also shown that CS inhibits the development of AS in rabbit models with RA [101,102].

CS effectively regulates blood lipid levels. In previous studies, chondroitin sulfate A decreases the lipid levels of serum and aortic in monkeys fed with a high-fat diet [103]. In addition, fucosylated CS extracted from sea cucumbers improves lipid profile, in which fucosylated CS with 3,4-O-disulfate fucose branches are much more effective in improving blood lipid and atherosclerosis index and could be potential in hypolipidemic treatment [104].

There is preliminary evidence showing that, in humans, CS may also be beneficial in inflammatory diseases such as AS [105,106]. Studies have found that CS relieves the inflammation in AS plaques, reduces the expression of VCAM-1, ICAM-1 and ephrin-B2 and improves the migration of inflamed endothelial cells and foam cell formation [98]. In addition, the administration of CS reduces the levels of C-reactive protein and IL-6 in serum, which is shown to be positively related to inflammation. Apart from that, CS inhibits the expression of CCL2/monocyte chemoattractant protein-1 and cyclooxygenase-2 (COX-2) in peripheral blood mononuclear cells and reduces the nuclear translocation of factor-kB. Moreover, CS improves the condition of aorta in rabbits with AS and chronic arthritis [102].

### 2.7. Chitosan

Chitosan is a deacetylated product of chitin, which is composed of β-(1→4)-2-acetylglucosamine and β-(1→4)-2-amino-d-glucose groups. Chitosan is the only basic polysaccharide in natural polysaccharides containing a large number of amino groups [107]. Widely distributed, chitosan exists in cell walls of some fungi, algae and the bones and shells of shrimp, crabs and squid pens [108]. Since chitosan carries a positive charge, it can bind negatively charged substrates such as lipids and fats when it is dissolved in an acidic environment [109]. For example, chitosan can be taken orally to bind fat in the intestine, increase the amount of fat in stool and reduce cholesterol in the body [110]. With continuous research on chitosan and its derivatives, the anti-atherosclerotic effects they show has attracted increasing attention.

As a dietary supplement, chitosan and its derivatives inhibit the formation of atherosclerotic plaques. Animals fed for 20 weeks on a diet containing 5% chitosan show lowered blood cholesterol levels and inhibition of atherogenesis in the aorta. This suggests that the agent could be used to inhibit the development of AS in individuals with hypercholesterolemia [109]. Chitosan oligosaccharide (COS) treatment attenuates AS, decreases plasma non-HDL level and aortic lesion and improves plaque condition in ApoE-/-mice. The potential mechanism may relate to the upregulation of gene expression of hepatic LDLR, SR-B1 and macrophage ATP-binding transporter A1 (ABCA1) [111,112].

Previous studies have shown that COS reduces the cholesterol content in the serum of mice and increases the number of white blood cells and the proportion of lymphocytes, indicating that COS may alleviate the effects of inflammation on AS by enhancing immune function [113]. Related mechanism studies have shown that COS suppresses VCAM-1 and ICAM-1 overexpression in endothelial cells through MAPKs and NF-κB, thereby preventing the damage of AS caused by vascular inflammation [113,114].

It is worth noting that modification of chitosan for the diagnosis and treatment of AS has become a hot topic in this field. Ye et al. successfully fabricated nanoparticles targeting SR-A, which is potential for the treatment of atherosclerosis. Moreover, in a vitro model of atherosclerotic plaque, it has shown the fact that the nanoparticles selectively accumulated at sites with high SR-A expression and the use of low intensity focused ultrasound-induced phase transition improves the condition of vessels in vivo [115]. Li et al. conjugated hydroxybutyl chitosan (HBC) to anti-CD133 antibody to prepare a CD133 antibody-coated stent, which has shown the ability to decrease intimal hyperplasia and reduce restenosis in contrast with bare stents, which suggests that the stents of the CD133 antibody coat may contribute to the treatment of AS [116]. In addition, implantation of sulfated chitosan into the perivascular compartment achieves therapeutic paravasal angiogenesis and inhibits the early signs of atherogenic inflammation [117]. These studies provide new research directions and treatment ideas for AS and stimulate further development of chitosan and its derivatives in this field.

### 2.8. Summary

In recent years, marine polysaccharides have become a hot research topic for marine-derived active substances. Marine polysaccharides come from rich natural sources and have limited toxic and side effects and diverse biological activities. At present, marine polysaccharides with anti-atherosclerotic effects can be roughly divided into two categories. Among them, polysaccharides derived from seaweeds are widely studied such as fucoidan, alginate, ulvan and *E. prolifera* polysaccharides. Another class of marine polysaccharides with anti-atherosclerotic effects are polysaccharides derived from marine animals such as chondroitin sulfate and chitosan. Among the polysaccharides discussed above, only fucoidan, chondroitin sulfate and chitosan directly hinder the development of AS and show the ability to decrease the area of plaques in animal models. Other polysaccharides such as ulvan and alginate reduce the risk factors for atherosclerotic progression by lowering blood lipids, improving antioxidant responses and suppressing inflammation, although there is no direct evidence that they can reduce the area of atherosclerotic plaques.

Commonly accepted, the bioactivities of polysaccharides depend on the molecular structure, which is influenced by the monosaccharide composition, glycosidic bond, substitution and branching, all contributed to the conformation [118]. Polysaccharides are characteristically complex structure and uneven. The structural identification of polysaccharides is still the focus of current research and poses many challenges. Most studies have only investigated the pharmacological activity of polysaccharides in AS and the relationship between the structure of polysaccharides and their anti-atherosclerotic effects and the mechanisms of action need to be further clarified. Therefore, we need to employ the rich marine resources to continue investigations on the structure and efficacy of polysaccharides to promote its development and utilization.

## 3. Proteins and Peptides

In recent years, scientists have successively found that sea cucumbers, jellyfish, scallops, seaweeds and other marine organisms have functions of lowering blood liquid, lowering blood glucose, antioxidant, immune regulation, protecting endothelial cells and so forth. Proteins and active peptides derived from marine organisms have received widespread attention. There are two main sources of marine bioactive peptides, one of which is the peptide inherent in marine organisms and the other is obtained by enzymatic hydrolysis of marine proteins. We summarize the effects of marine proteins and peptides on AS in Table 4.

Studies have reported that marine bioactive peptides regulate blood lipids, thereby preventing the development of AS. One study found that *porphyra peptide* effectively inhibits the increase in body weight of hyperlipidemia rats and reduces serum TC, TG and LDL-C while increasing HDL-C and lowering the atherosclerosis index [119]. In addition, treatment of rats with peptides from sea cucumber increases serum HDL-C and reduces TG levels [120]. *Salmon protein hydrolysate* (SPH) is believed to improve lipid metabolism and inflammation, all contributed to the anti-atherosclerotic properties [124]. Administration of SPH to ApoE-/-mice reduces the area of the atherosclerotic plaques in the aorta accompanied by decreases in the inflammatory factors IL-1β, IL-6, TNF-α, granulocyte-macrophage colony-stimulating factor (GM-CSF) and granulocyte colony-stimulating factor (G-CSF) in plasma [124].

Protecting vascular endothelial cells is also beneficial to prevent the development of AS [125]. It has been demonstrated that sea cucumber collagen peptides reduce the MDA level, inhibit the production of lipid peroxide, improve the activity of NO synthase, promote the production of NO and maintain the normal physiological function of vascular endothelial cells [126].

In summary, the world has an abundance of marine protein resources which has broad therapeutic prospects. Although many studies have been conducted on marine bioactive peptides, the utilization rate remains low. The studies mostly concentrate on a few functional peptides with anti-oxidant, anti-bacterial or anti-hypertensive properties while investigations on hypolipidemic peptides are lacking in comparison. In addition, most of the studies concerning anti-atherosclerotic marine peptides do not examine the relationship between the peptide structure and amino acid composition and activity as well as the underlying mechanisms of action. More comprehensive and systematic reports are needed in these areas further develop marine-derived bioactive peptides.

## 4. Polyunsaturated Fatty Acids

Polyunsaturated fatty acids (PUFAs) refer to fatty acids with multiple unsaturated bonds in the carbon chain. They are mainly divided into ω-3 PUFAs and ω-6 PUFAs according to the position of the double bond. ω-3 PUFAs are mainly composed of Eicosapentaenoic Acid (EPA), Docosahexaenoic Acid (DHA) and α-linolenic acid (ALA). EPA and DHA are abundant in marine organisms such as fish, marine mammals and seaweed while ALA is mainly derived from plants [127]. Findings indicate that Alaska natives with marine fish, whales, seals and other marine animals as the main food source had less advanced atherosclerosis in coronary arteries than non-indigenous people [128]. These findings suggest that EPA and DHA derived from fish oil have potential preventive and therapeutic properties for treating AS.

ω-3 PUFAs reduce atherosclerotic plaque areas and enhance plaque stability. In one study, patients treated with EPA showed a reduction in atherosclerotic plaque area and thickening of the plaque fiber cap [129]. Studies conducted in patients undergoing carotid endarterectomy showed that ω-3 PUFAs is rapidly incorporated into advanced atherosclerotic plaques, which increases their stability [130]. Atherosclerotic plaques are more stable with higher EPA intake in patients with carotid plaques [131].

Endothelial dysfunction is an early event that occurs in AS [132]. iNOS expression level is closely related to endothelial cell function. During endothelial dysfunction, NO release is reduced or abrogated. Many studies have shown that ω-3 PUFAs improve normal or impaired endothelial function through different mechanisms, which has an inhibitory effect on AS progression [133,134,135]. ω-3 PUFAs increase the effectiveness of NO by activating NOS, which may be the most important effect of ω-3 PUFAs on endothelial cells [136]. Studies have shown that both EPA and DHA affect the micro-environment of caveolae, which redistributes the NOS to the surface of the cell membrane, leading to its activation and increases the synthesis of NO [137,138,139].

High TG levels are associated with an increased risk of cardiovascular disease and ω-3 PUFAs regulate blood lipids by reducing TG. It has been shown that the increase in TG-rich lipoproteins such as V-LDL and chylomicrons promote the development of AS and other cardiovascular diseases [140]. Clinical studies have shown that the combined use of ω-3 PUFAs and statins further reduce TG content [138]. In 47 randomized controlled trials, it was found that ω-3 PUFAs have the ability to lower fasting TG levels in a dose-dependent manner and the effect of these compounds is related to EPA and DHA intake and initial TG levels [137]. In addition, ω-3 PUFAs also change the volume of lipoprotein particles. Supplementing DHA increases the volume of HDL particles, which allows HDL to carry more cholesterol and ultimately improves blood lipid levels [141,142,143].

AS is a chronic inflammatory disease and the anti-inflammatory effects of ω-3 PUFAs counteract the occurrence and development of AS. Human inflammatory cell membranes are usually rich in arachidonic acid (AA). AA mainly plays an important part in the inflammatory cell membrane as a precursor of eicosanoid including various pro-inflammatory factors such as prostaglandins (PG), thromboxanes (TX) and leukotrienes (LT) [144]. Studies have found that ω-3 PUFAs compete to replace AA during metabolism, thereby reducing PG, TX, LT and other inflammatory factors produced by AA [127,145]. Patients given EPA treatment before carotid plaque resection surgery showed weakened inflammatory response in the carotid plaques and a reduction in the number of T cells. Moreover, the transcription levels of IL-6 and ICAM-1 are also reduced [131]. Other studies have demonstrated similar anti-inflammatory properties of ω-3 PUFAs, which is shown to have the ability to a decrease the levels of some pro-inflammation cytokines such as TNF-α, IL-1β and C-reactive protein [146,147,148,149].

Monocyte-macrophages are the primary immune cells involved in the initiation and development of AS. Changes in macrophage phenotype have been observed during the development of AS [150] and supplementation of DHA help macrophages to transform from an M1 proinflammatory to an M2 repair type [151]. However, whether this transformation has a positive effect on AS requires further study.

In summary, ω-3 PUFAs not only contain essential fatty acids but also perform various regulatory roles such as protecting endothelial function and moderating blood lipid levels, inflammation and immunity. Overall, fish oil-derived ω-3 PUFAs show great potential for the treatment of AS. It has been shown that ω-3 PUFAs play a role in inhibiting the risk factors of AS.

## 5. Small Molecule Compounds

### 5.1. Astaxanthin

**Figure 5:**
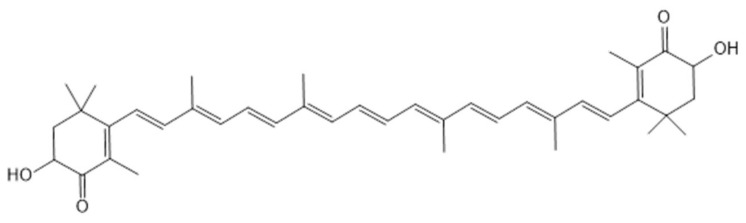
The structure of Astaxanthin [154].

Astaxanthin is a naturally occurring red carotenoid pigment that is widely found in marine organisms such as shrimp, crab, salmon and microalgae (Figure 5) [152,153]. Accumulating evidence suggests that astaxanthin could prevent or treat AS and cardiovascular diseases by lowering blood lipids, antioxidant effects, improving inflammation and regulating glucose metabolism [153].

Astaxanthin treatment reduces the occurrence of AS in experimental animal models. In one study, astaxanthin was given to rats fed with a high-cholesterol diet, which results in a reduction in TC, TG, LDL-C and VLDL-C and an increase in HDL-C. Furthermore, the number of foam cells and the area of atherosclerotic plaques in the aorta are reduced by astaxanthin [155]. Similar findings have been reported in rabbits where astaxanthin improve plaque stability by decreasing macrophage infiltration and apoptosis [156]. The novel astaxanthin prodrug CDX-085 lowers TC and aortic arch AS in LDLR-/-mice and TG in ApoE-/-mice [157]. Yang et al. report that after exposure to astaxanthin, mRNA levels of LDLR, 3-hydroxy-3-methylglutaryl CoA reductase and SREBP-2 is increased. This shows that the hypolipidemic effect of astaxanthin may be contributed by the upregulation of LDLR, which helps to mitigate the progression of AS [158]. In addition, astaxanthin promotes reverse cholesterol transport and regulates plasma cholesterol levels in ApoE-/-mice [159].

The antioxidant activity of astaxanthin has been demonstrated in several studies. In one study, astaxanthin pre-treatment inhibits homocysteine-induced cytotoxicity and increases human umbilical vascular endothelial cells migration, invasion and tube formation. The mechanism may involve the effective inhibition of homocysteine-induced ROS generation and the recovery of focal adhesion kinase phosphorylation [160]. Similarly, astaxanthin can reduce homocysteine-induced H9c2 cytotoxicity in the mechanism of downregulating mitochondrial-mediated apoptosis as well as reducing ROS to improve oxidative damage [161]. The antioxidant capacity of astaxanthin has also been confirmed in animal studies. Atherosclerotic rabbits show increased aortic lipid peroxidation and nonprotein thiol group levels, which is attenuated by astaxanthin by increasing the activity of aortic SOD, CAT and thioredoxin reductase activities [162]. In addition, oral administration of astaxanthin in diabetic rats decreases the level of MDA in the aorta and the degree of LDL oxidation [163]. Study on the relationships between the structure of astaxanthin and antioxidant activity have shown that there are many conjugated double bonds in the molecular structure of astaxanthin. The end of the conjugated double bond chain has unsaturated ketone groups and hydroxyl groups that form an α-hydroxy ketone. This charged structure allows astaxanthin to easily react with free radicals and plays a significant role in anti-oxidation [164].

In previous study, astaxanthin significantly inhibited the expression of the SR-A and CD36, the activity and the expression of matrix metalloproteinases and the mRNA expression of various inflammatory mediators including TNF-α, IL-1β, IL-6, iNOS and COX-2 in THP-1 macrophages [165]. In another study, astaxanthin inhibited the activation of IκB-dependent NF-κB in RAW264.7 macrophages and primary macrophages stimulated by LPS and reduced the expression of pro-inflammatory mediators such as prostaglandin E2, TNF- α and IL-1β [166]. In addition, Astaxanthin enhanced the phagocytic and bactericidal ability of neutrophils and inhibited the production of superoxide anions and hydrogen peroxide, which may be mediated by the production of calcium and NO released from intracellular storage [167]. These studies indicate that astaxanthin has anti-inflammatory effects by inhibiting NF-κB activation, which may be related to its antioxidant activity.

### 5.2. Sponge Extract

Among marine organisms, sponges are a unique group with abundant secondary metabolites. Studies have shown that secondary metabolites isolated from sponges have the potential to treat AS.

**Figure 6:**
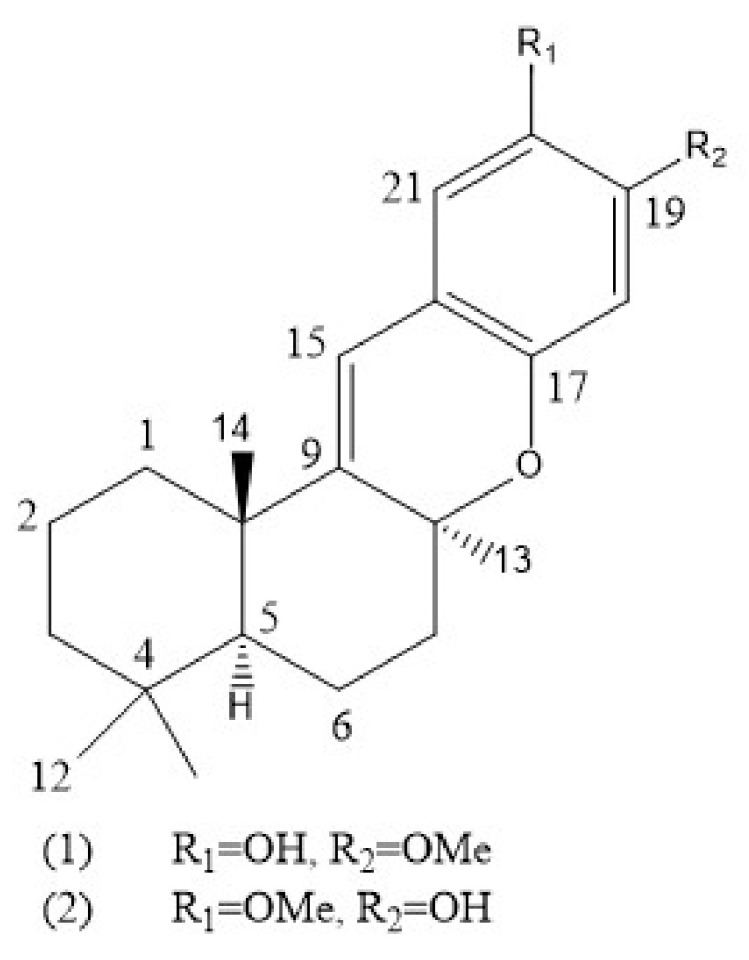
The structures of compounds 1 and 2 isolated from sponge *Hyrtios digitatus* [171].

SR-B1 is a receptor for HDL, which plays an important role in the development of AS [168,169]. HDL can mediate reverse cholesterol transport by binding to SR-B1 on the cell surface, thereby reducing the risk of AS [170]. Tetracyclic merosesquiterpene is isolated from the Australian sponge *Hyrtios digitatus*. After further purification, 19-methoxy-9,15-ene-puupehenol and 20-methoxy-9,15-ene-puupehenol are obtained (Figure 6), which upregulate the activity of SR-B1 in HepG2 cells in a dose-dependent manner [171]. In another report, aaptamine (1), 9-demethylaaptamine (2), 4-*N*-methylaaptamine (3), 9-methoxyaaptamine (4), 9-demethyloxyaaptamine (5), 4-hydroxybenzamide (6) and 3β,5α-cholesterol (7) has been isolated from the *Aaptos aaptos* sponge [172]. Among them, compounds 1 – 4 and 7 increase the transcription level of SR-B1 and PPAR response element (PPRE) in HepG2 cells, which is the binding site of PPARγ in the SR-B1 promoter region. The combination of PPRE and PPARγ promote the expression of SR-B1 [173]. This indicates that sponge extracts may exert anti-atherosclerotic effects through PPRE and SR-B1. However, there are limited reports on the treatment of AS with sponge extract and further research is needed to explore new compounds from sponges that have a positive effect on AS.

### 5.3. Sea Cucumber Saponins

**Figure 7:**
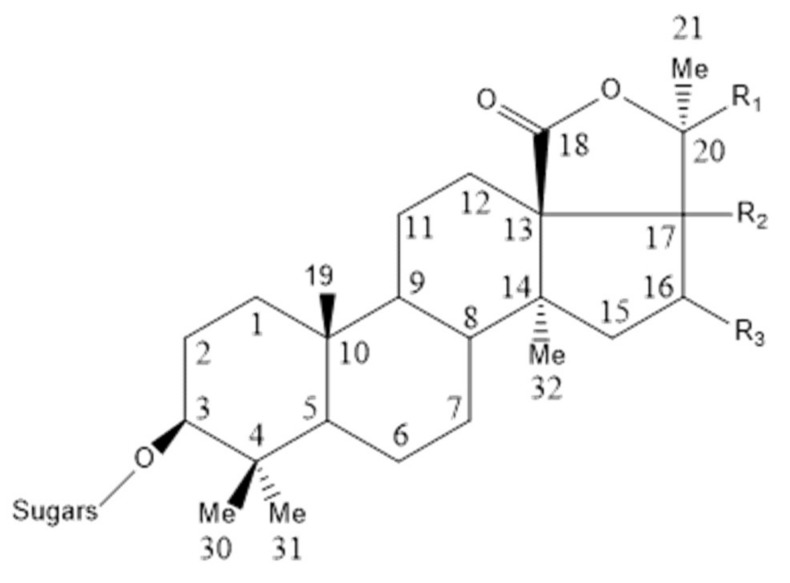
Basic structure of holostane saponins extracted from sea cucumber [178].

Sea cucumber saponins (SCSs) are the main secondary metabolite of sea cucumbers and are also the material basis for the chemical defense of sea cucumbers. SCSs are a class of glycosides whose aglycones are triterpene or spirostane compounds [174,175]. The aglycones and sugar chains of SCSs are primarily connected by β-glycosidic bonds (Figure 7) [176,177]. The molecular weight of aglycone chelate is 500–1500 Da, which is typically composed of 30 carbon atoms [178]. Recent studies have shown that SCSs have a significant role in improving lipid metabolism and preventing AS.

Studies have shown that SCSs indirectly clear atherosclerotic plaques. In ApoE-/-mice model fed with a cholesterol-rich diet, treatment with SCS reduces the levels of serum lipid in a dose-dependent manner and promotes plaque regression [179]. *Thelenota ananas* saponin extract also reduces the aortic plaque area in ApoE-/-mice by exerting anti-inflammatory and improving blood lipid effects [180].

The anti-atherosclerotic role of SCSs is attributable to its ability to regulate lipid metabolism. Studies have shown that *T. ananas* saponin extract holothurin A (desHA) has a significant regulatory effect on cholesterol metabolism in foam cells induced by ox-LDL. This is because desHA regulates the concentration of HMG-CoA reductase and NOS by blocking the LXR/AKT/AMPK pathway. Thus, desHA plays an important role in inhibiting intracellular cholesterol synthesis and promoting intracellular cholesterol outflow [181]. In addition, SCSs reduce the levels of TG, TC and LDL-C in serum of mice and rats [179,182,183] and increase the levels of HDL-C [181].

Inflammation is centrally involved throughout the pathogenesis of AS. Studies have shown that SCSs possess anti-inflammatory capabilities by lowering TNF-α in the serum of ApoE-/-mice and downregulating TNF-α mRNA expression the aorta [179]. In addition, SCSs inhibit the production of inflammatory factors such as IL-1β, IL-6 and MCP-1 [180,184]. In conclusion, SCSs exhibit effective therapeutic effects on AS and may be a promising active ingredient in novel AS therapy. The precise mechanisms of SCSs is to be determined, which means further research is still needed.

### 5.4. Asperlin

**Figure 8:**
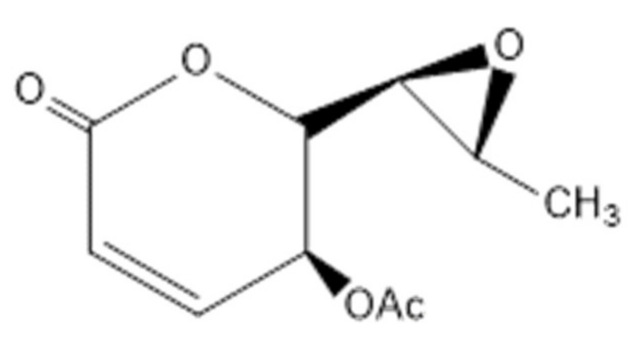
The structure of Asperlin [186].

Asperlin is a fungal metabolite which was first isolated from the marine-derived *Aspergillus sp. SF-5044* (Figure 8) [185]. It has been shown to exhibit anti-tumor, anti-bacterial, anti-fungal and anti-inflammatory properties [185,186,187,188], which may be conducive to preventing the development of AS [186].

Researchers have found that asperlin from marine *Aspergillus versicolor LZD4403* has the ability to inhibit the formation of foam cells induced by LPS as well as promote cholesterol efflux in RAW264.7 macrophages. Animal experiments showed that oral administration of asperlin remarkably inhibits the formation of atherosclerotic plaque in the aorta and reduces the lesion area [186]. In addition, asperlin decreases serum levels of pro-inflammation cytokines such as MCP-1, TNF-α and IL-6, although the concentration lipid profiles were unchanged. This indicates that asperlin may play an anti-atherosclerotic effect by suppressing inflammation rather than improving dyslipidemia.

### 5.5. Mycoepoxydiene

**Figure 9:**
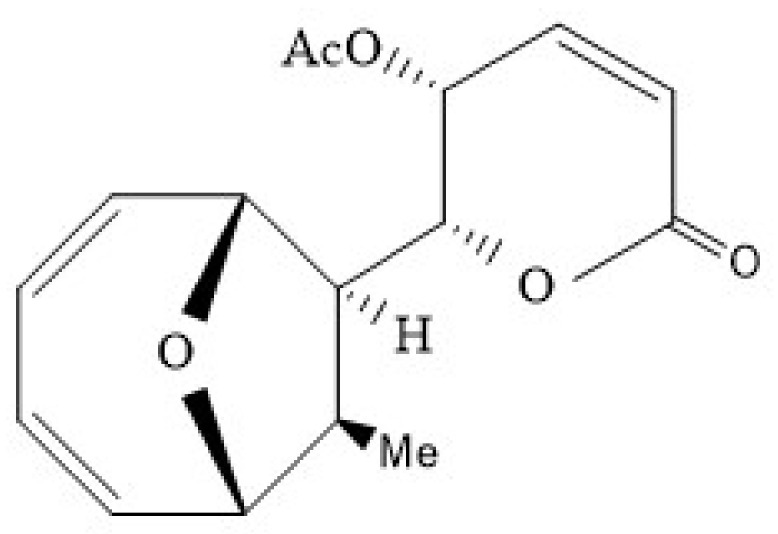
The structure of Mycoepoxydiene [191].

A polyketide named mycoepoxydiene (MED) can be obtained from the marine fungus *Diaporthe sp. HLY-1* [189,190], which contains an α,β-unsaturated-lactone moiety and an oxygen-bridged cyclooctadiene core (Figure 9) [191]. Previous studies have shown that MED exerts anti-microbial, anti-tumor, anti-anaphylactic and anti-inflammatory effects [189,192,193,194]. Recent studies suggest that MED may have the potential for the development of lead compound in the treatment of atherosclerosis [195].

MED inhibits LPS-induced inflammatory responses in mice and the expression of pro-inflammatory mediators such as TNF-α, IL-6, IL-1β and NO in the mechanism of deactivation of both NF-κB and MAPK pathways [192]. Furthermore, MED is shown to prevent the foam cell formation induced by the ox-LDL and suppress the gene expression of LOX-1, which is responsible for binding and uptake of ox-LDL in endothelial cells [195,196]. Apart from that, MED surprisingly improves the high-fat diet-induced atherosclerosis in ApoE-/- and LDLR-/-mice [195,197].

### 5.6. Xyloketal B

**Figure 10:**
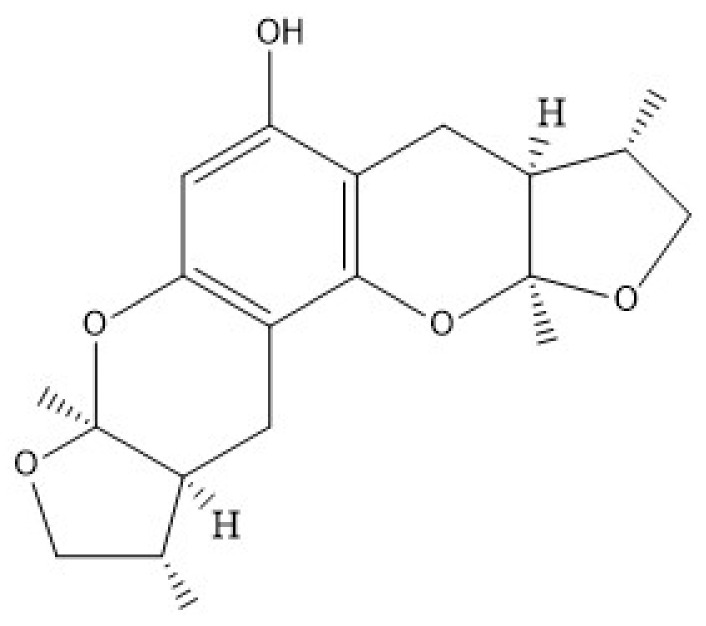
The structure of Xyloketal B [198].

Isolated from *Xylaria sp. 2508* in the South China Sea, Xyloketal B is a novel marine compound with a unique chemical structure (Figure 10) [198]. The accumulating evidence suggests that xyloketal B has strong antioxidant actions. Xyloketal B can directly decrease free radical and ROS as well as protect human umbilical vein endothelial cells from ox-LDL-induced damage [199,200]. In addition, xyloketal B also shows good endothelial protection, which augment the production and bioavailability of NO [200,201]. Due to its special antioxidant properties and effects in endothelial protection, xyloketal B is expected to be used in the treatment of AS.

In a recent study, Xyloketal B dose-dependently reduced the area of atherosclerotic plaque in the aortic sinus and the entire aorta of mice fed high-fat diet. Furthermore, xyloketal B significantly reduces the levels of vascular oxidative stress, improves the damaged endothelium integrity and NO-dependent aortic vasorelaxation in atherosclerotic mice [202]. Apart from that, other studies have got the fact that xyloketal B decreases serum LDL-C, showing that xyloketal B may have a beneficial impact on the different aspects of AS [203].

### 5.7. Summary

Marine organisms are rich in species and the active substances extracted from marine organisms have great potential value for research and clinical application. Small molecules obtained from marine-derived sources have novel structures and beneficial biological activities, which are valuable resources for lead compounds of natural marine drugs. In this chapter, we mainly described astaxanthin, sponge extracts, SCSs and some secondary metabolites of marine fungi. Among them, astaxanthin has a strong antioxidant capacity and is promising in preventing diseases related to oxidative stress [204]. Recent studies have shown that astaxanthin reduces the area of atherosclerotic plaques in animals and exert anti-atherosclerotic effects by improving oxidative stress and lipid metabolism. Sponge extracts and sea cucumber saponins show potential in regulating blood lipid levels. In many studies on secondary metabolites of marine fungi, only a small number of compounds have been found to exhibit positive therapeutic effects on AS. After treatment with asperlin, MED or xyloketal B, the atherosclerotic plaque areas of mice are significantly decreased. Among them, asperlin and MED may reduce the levels of pro-inflammation cytokines in the serum of mice and inhibit the inflammatory response, thereby dampening AS progression. The anti-atherosclerotic effects of xyloketal B can be attributed to the properties of anti-oxidation and endothelial-protection.

With further study on marine-derived small molecules, more novel biologically active extracts will be discovered. The elucidation of the structure, pharmacological activity and mechanisms of action of the new compounds is crucial for advancing our search for marine drugs for the treatment of AS.

## 6. Concluding Remarks and Future Outlooks

As an important part of marine active substances, polysaccharides have shown promising biological activity in the treatment of AS. In this review, we highlight the therapeutic effects of marine polysaccharides on AS by regulating blood lipid metabolism, anti-inflammatory and anti-oxidant abilities. It is worth noting that marine polysaccharides obtained from different species and different extraction methods have different functions and activities as well as a high degree of heterogeneity which makes it challenging for the study of marine polysaccharides [5]. However, the high degree of heterogeneity has also brought challenges to the structural studies of marine polysaccharides. Algal polysaccharides usually have a high molecular weight, which results in low oral bioavailability. The existence of these problems may hinder their development as therapeutic agents. In addition, in the study of marine polysaccharides for the treatment of AS, most of the studies used a mixture of polysaccharides, which is unfavorable for investigating the mechanisms of action. Therefore, future research needs to improve the purity of the sample and conduct in-depth research on its structure.

Marine bioactive peptides have broad prospects due to their unique advantages such as diverse functions, wide-spread sources, strong specificity and low toxicity and side effects. However, the abundant availability of marine bioactive peptides has not brought breakthrough discoveries for the treatment of AS. Most of the current research focuses on marine bioactive peptides by regulating blood lipid metabolism and protection of endothelial cells to play an active therapeutic effect on AS and research in this area is also limited. In addition, the influence of the amino acid sequence and structure of marine peptides on the specific mechanisms of its effect needs further study.

The most important structural characteristic of marine lipids is the richness in polyunsaturated fatty acids. It has been demonstrated that ω-3 PUFAs are efficacious in preventing cardiovascular and cerebrovascular diseases. It can be used as a potential treatment for AS by regulating blood lipids and exerting anti-inflammatory and protective effects on endothelial cells.

Small molecules such as astaxanthin, SCSs and some secondary metabolites of marine fungi have a novel structure and beneficial biological activity. In many studies on marine small molecules, only a limited number of compounds have shown positive therapeutic effects on AS. Therefore, continued exploration and discovery will provide broader ideas for the development of this field.

In conclusion, there are many marine substances with the potential of treating atherosclerosis while the current researches are still at an early stage, which are mainly based on the cell and animal experiments. As present, the mechanisms of bioactive substances discussed above are still to be determined, which limits the further development. In addition, as is shown in the research of marine polysaccharides and peptides, the current dosage of animal experiments is quite high, which is mainly caused by the relatively lower oral utilization. Different ways of drug delivery and structural modifications may contribute to the better usage of these substances, which will greatly promote the treatment of atherosclerosis.

## Figures and Tables

**Table 1:** The composition and effect of fucoidans.

Activities	Sources	Composition	Indices Level (↑: Up-Regulation; ↓: Down-Regulation)	Reference
Lipid-lowering	*S. japonica*	molecular weight = 8177Da, fucose 35.07%, sulfate 36.85% and uronic acid 0.039%.	↓ TC*, TG*, LDL-C*, ox-LDL* ↑ HDL-C*	[13]
	*S. japonica*	molecular weight = 8177Da, fucose 35.07%, sulfate 36.85% and uronic acid 0.039%	↓ TG, ox-LDL ↑ HDL-C	[14]
	*Cladosiphono* *Kamuranus Tokida*	glucuronic acid 6.7% ± 0.5%, fucose 36.2% ± 3.5%, galactose 1.0% ± 0.3%, mannose 1.0% ± 0.4%, glucose 0.5% ± 0.08% and xylose 1.9% ± 0.7%	↓ TC, TG, LDL-C ↑ HDL-C, LPL*	[15]
	*F. vesiculosus* *(Sigma-Aldrich)*	-	↓ TC, TG, LDL-C ↑ HDL-C	[17]
	*A. nodosum*	molecular weight = 361.4 kDa, carbohydrate 68.0%, sulfate 16.6% and protein 2.8%	↓TC, TG, LDL-C, ApoB* ↑ApoA1*, LPL	[22]
	*L. japonica*	molecular weight = 189kDa, total sugar 48%, fucose 29% and sulfate 28%	↓ TC, TG, LDL-C ↑ HDL-C	[25]
	*L. japonica*	molecular weight = 76 kDa, fucose 59.85%, arabinose 7.89% and rhamnose 0.14%	↓ TC, TG, LDL-C ↑ HDL-C, LPL, HL*, LCAT*	[19]
	*A. nodosum*	molecular weight =207.2kDa, carbohydrate 60.4%and sulfate 16.6%	↓ TC, TG ↑ HDL-C	[23]
Anti-inflammatory	*S. japonica*	molecular weight = 8177Da, fucose 35.07%, sulfate 36.85% and uronic acid 0.039%	↓ IL*-6, IL-10, p-SAPK*	[13]
	*S. japonica*	molecular weight = 8177Da, fucose 35.07%, sulfate 36.85% and uronic acid 0.039%	↓ IL-6, IL-10, p-JNK*, cyclin D1,	[14]
	*L. japonica*	molecular weight = 189kDa, total sugar 48%, fucose 29% and sulfate 28%	↓ IL-1β, IL-6, TNF-α*, ICAM-1*, VCAM-1*	[25]
Antioxidant	*F. vesiculosus*	molecular weight = 160 kDa, fucose 88.4%, galactose 6.0% and xylose 1.8%	↓ MDA*, DC*	[18]
	*L. japonica*	molecular weight = 189kDa, total sugar 48%, fucose 29% and sulfate 28%	↓NOX-2*, NOX-4*, eNOS*, SOD1*	[25]

* TC: total cholesterol; TG: triacylglycerol; LDL-C: low-density lipoprotein cholesterol; ox-LDL: oxidized low-density lipoprotein; HDL-C: high-density lipoprotein cholesterol; ApoB: Apolipoprotein B; ApoA1: Apolipoprotein A1; LPL: lipoprotein lipase; HL: hepatic lipoprotein; LCAT: lecithin cholesterol acyltransferase; IL: Interleukin; p-SAPK: stress activated protein kinase; p-JNK: phosphorylated c-Jun N-terminal kinase; TNF-α: tumor necrosis factor-alpha; ICAM-1: intercellular cell adhesion molecule-1; VCAM-1: vascular cell adhesion molecule-1; MDA: malondialdehyde; DC: diene conjugates NOX-2/4: NADPH oxidase subunit 2/4; eNOS: endothelial nitric oxide synthase; SOD1 superoxide dismutase 1.

**Table 2:** The composition and effect of ulvan.

Activities	Sources	Composition	Indices Level (↑: Up-Regulation; ↓: Down-Regulation)	Reference
Lipid-lowering	*Ulva lactuca*	rhamnose, galactose, glucose, arabinose, xylose, mannose glucuronic acid and galacturonic acid	↓Total lipids, TC, TG, LDL-C, VLDL-C ↑ HDL-C	[71]
	*Ulva pertusa* *(Chlorophyta)*	ronic acids, rhamnose, xylose, glucose and sulfate comprised their main composition, with smaller amounts of mannose, arabinose and galactose. basic repeating units of the polysaccharides were (β-d-GlcpA-(1→4)-α-l-Rhap 3S) and (α-l-IdopA-(1→4)-α-l-Rhap 3S)	↓ TC, TG, LDL-C ↑ HDL-C	[72]
	*Ulva pertusa* *(Chlorophyta)*	Ulvan molecular weight = 151.6 kDa, total sugar 47.6%, sulfate 17.1% and uronic acid 23.2%	↓ TC, LDL-C	[76]
		U1 molecular weight = 64.5 kDa, total sugar 47.8%, sulfate 16.8% and uronic acid 22.7%	↓ TG ↑ HDL-C	
		U2 molecular weight = 28.2 kDa, total sugar 48.1%, sulfate 17.4% and uronic acid 23.0%	↓ TG ↑ HDL-C	
Antioxidant	*F. vesiculosus*	molecular weight = 160 kDa, fucose 88.4%, galactose 6.0% and xylose 1.8%	↓ TBARS* ↑ CAT*, GSH-Px*, SOD*, GSH*, T. thiol*	[71]
	*Ulva pertusa Kjellm (Chlorophyta)*	Ulvan molecular weight = 151.7 kDa, neutral sugar 25.6%, sulfate 19.9% and uronic acid 19.2%	Hydroxyl radical scavenging activities U3 > U1(U) > U2	[77]
		U1 molecular weight = 64.5 kDa, neutral sugar 24.8%, sulfate 20.4% and uronic acid 18.9%		
		U2 molecular weight = 58.0 kDa, neutral sugar 26.3%, sulfate 19.1% and uronic acid 20.1%		
		U3 molecular weight = 28.2 kDa, neutral sugar 25.1%, sulfate 19.4% and uronic acid 19.0%		

* TBARS: thiobarbituric acid reactive species; CAT: liver catalase; GSH-Px: glutathione peroxidase; GSH: hepatic reduced glutathione; SOD: superoxide dismutase; T. thiol: total thiol.

**Table 3:** The composition and effect of *E. prolifera* polysaccharides.

Activities	Sources	Composition	Indices Level (↑: Up-Regulation; ↓: Down-Regulation)	Reference
Lipid-lowering	*E. prolifera*	EPF2* molecular weight = 103.51 kDa, carbohydrates 53.2%, proteins 11.5%, sulfate group 18.6% and uronic acid 12.4%; rhamnose, xylose, mannose, galactose and glucose in a molar ratio of 3.64:1.08:0.21:0.75:0.27.	↓ TC, TG, LDL-C ↑ HDL-C	[78]
	*E. prolifera*	EPsmolecular weight = 134.07 kDa, total sugar 54.6%, protein 10.1%, uronic acid 12.4% and sulfate contents 17.9%; rhamnose, xylose, mannose, galactose and Glucose	↓ TC, TG, LDL-C ↑ HDL-C	[81]
	*E. prolifera*	rhamnose, glucuronic acid, arabinose, fucose, xylose and glucose in a molar ratio of 5.12:1.32:3.38:1.62:1:1.03.	↓ TG, HMGCR, SREBP-2	[82]
	*E. prolifera*	rhamnose, glucuronic acid, arabinose, fucose, xylose and glucose in a molar ratio of 5.12:1.32:3.38:1.62:1:1.03.	↓ TG, ACC, SREBP-1c	[83]
Antioxidant	*E. prolifera*	EPF2 molecular weight = 103.51 kDa, carbohydrates 53.2%, proteins 11.5%, sulfate group 18.6% and uronic acid 12.4%; rhamnose, xylose, mannose, galactose and glucose in a molar ratio of 3.64:1.08:0.21:0.75:0.27.	↓ MDA ↑ CAT, GSH-Px, SOD	[78]

* EPF2: A polysaccharide fraction was obtained from the crude polysaccharides of *E. prolifera.*

**Table 4:** The effects of marine proteins and peptides.

Activities	Sources	Indices Level (↑: Up-regulation; ↓: Down-regulation)	Reference
Lipid-lowering	*Porphyra peptide*	↓ TC, TG, LDL-C, LDL-C/HDL-C ↑ HDL-C	[119]
	*Holothuria forskali protein*	↓ TG ↑ HDL-C	[120]
	*Scallop skirt peptide*	↓ TC, TG, LDL-C ↑ HDL-C	[121]
	*Jellyfish collagen peptide*	↓ TC, TG, LDL-C, LDL-C/HDL-C ↑ HDL-C, HDL-C/TC	[122]
	*Pearsonothuria graef feihomogenate*	↓ TC, LDL-C, LDL-C/HDL-C ↑ HDL-C	[123]
	*Apostichopus japonicus feihomogenate*	↓ TC, LDL-C, LDL-C/HDL-C ↑ HDL-C	
Anti-inflammatory	*Salmon protein hydrolysat*	↓ IL-1β, IL-6, TNF-α, *GM-CSF, *G-CSF	[124]
Antioxidant	*Porphyra peptide*	↓ MDA ↑ GSH-Px, SOD	[119]
	*Scallop skirt peptide*	↓ MDA ↑ GSH-Px, SOD	[121]
	*Jellyfish collagen peptide*	↓ MDA ↑ GSH-Px, SOD	[122]
	*Pearsonothuria graef feihomogenate*	↓ MDA ↑ GSH-Px	[123]
	*Apostichopus japonicus feihomogenate*	↓ MDA ↑ GSH-Px, SOD	

* granulocyte-macrophage colony-stimulating factor (GM-CSF) and granulocyte colony-stimulating factor (G-CSF).
